# The *Mycobacterium avium* ssp. *paratuberculosis* specific *mpt*D gene is required for maintenance of the metabolic homeostasis necessary for full virulence in mouse infections

**DOI:** 10.3389/fcimb.2014.00110

**Published:** 2014-08-14

**Authors:** Thorsten Meiß, Elke Eckelt, Tina Basler, Jochen Meens, Julia Heinzmann, Abdulhadi Suwandi, Walter M. R. Oelemann, Sandra Trenkamp, Otto Holst, Siegfried Weiss, Boyke Bunk, Cathrin Spröer, Gerald-F. Gerlach, Ralph Goethe

**Affiliations:** ^1^Department of Infectious Diseases, Institute for Microbiology, University of Veterinary Medicine HannoverHannover, Germany; ^2^Helmholtz Centre for Infection Research, Molecular ImmunologyBraunschweig, Germany; ^3^Departamento de Imunologia, Instituto de Microbiologia Paulo Góes, Universidade Federal do Rio de Janeiro (UFRJ)Rio de Janeiro, Brazil; ^4^Division of Structural Biochemistry, Research Center Borstel, Leibniz-Center for Medicine and BiosciencesBorstel, Germany; ^5^Metabolomic Discoveries GmbHPotsdam-Golm, Germany; ^6^Bioinformatics, Leibniz Institute DSMZ-German Collection of Microorganisms and Cell CulturesBraunschweig, Germany; ^7^German Centre of Infection Research, Partner Site Hannover-BraunschweigBraunschweig, Germany

**Keywords:** Mycobacterium infections, metabolism, lipids, macrophages, survival

## Abstract

*Mycobacterium avium* subspecies *paratuberculosis* (MAP) causes Johne's disease, a chronic granulomatous enteritis in ruminants. Furthermore, infections of humans with MAP have been reported and a possible association with Crohn's disease and diabetes type I is currently discussed. MAP owns large sequence polymorphisms (LSPs) that were exclusively found in this mycobacteria species. The relevance of these LSPs in the pathobiology of MAP is still unclear. The *mpt*D gene (MAP3733c) of MAP belongs to a small group of functionally uncharacterized genes, which are not present in any other sequenced mycobacteria species. *mpt*D is part of a predicted operon (*mpt*ABCDEF), encoding a putative ATP binding cassette-transporter, located on the MAP-specific LSP14. In the present study, we generated an *mpt*D knockout strain (MAPΔ*mpt*D) by specialized transduction. In order to investigate the potential role of *mpt*D in the host, we performed infection experiments with macrophages. By this, we observed a significantly reduced cell number of MAPΔ*mpt*D early after infection, indicating that the mutant was hampered with respect to adaptation to the early macrophage environment. This important role of *mpt*D was supported in mouse infection experiments where MAPΔ*mpt*D was significantly attenuated after peritoneal challenge. Metabolic profiling was performed to determine the cause for the reduced virulence and identified profound metabolic disorders especially in the lipid metabolism of MAPΔ*mpt*D. Overall our data revealed the *mpt*D gene to be an important factor for the metabolic adaptation of MAP required for persistence in the host.

## Introduction

*Mycobacterium avium* subspecies *paratuberculosis* (MAP) belongs to the *M. avium* complex (MAC), which comprises subspecies of *M. avium* with different extents of host adaptation and virulence. For instance *M. avium* subsp. *hominissuis* is found ubiquitously in the environment and causes opportunistic infections in humans, pigs and ruminants, whereas *M. avium* subsp. *avium* is the causative agent of tuberculosis in birds (Thorel et al., 1990; Mijs et al., 2002; Dvorska et al., 2003; Dhama et al., 2011; Ignatov et al., 2012). In contrast, MAP is an obligate pathogen for ruminants causing Johne's disease (JD, paratuberculosis) - a chronic granulomatous enteritis (Kreeger, 1991; Harris and Barletta, 2001). Moreover, infections of humans with MAP have been reported and a possible association with Crohn's disease and more recently with diabetes type I has been discussed (Greenstein, 2003; Feller et al., 2007; Mendoza et al., 2009; Rani et al., 2010; Rosenfeld and Bressler, 2010; Cossu et al., 2011; Chiodini et al., 2012; Naser et al., 2014).

JD is characterized by an extending transmural inflammation of the intestine without caesification of the granulomas. Noteworthy, lesions in other areas are less common, indicating a specific tropism of MAP for the intestine which is not seen in other mycobacteria (Buergelt et al., 1978; Clarke, 1997; Burrells et al., 1998). The intestinal tropism of MAP is particularly evident in ruminants, but it has become apparent that the organism has a much broader host range including monogastric species such as carnivore (fox, stoats), aves (crow and jackdaw), lagomorpha (rabbits) and recently miniature donkeys (Greig et al., 1999; Beard et al., 2001; Glanemann et al., 2008; Stief et al., 2012; Carta et al., 2013).

The pathobiology of MAP infection including its tropism to the gut is still unresolved. After crossing the intestinal barrier, MAP is taken up by intestinal macrophages and there is common consensus that the persistence in these macrophages is the key step in MAP infection (Ryan et al., 2014). Nevertheless, the persistence in macrophages, characterized by inhibition of the phagosomal maturation process, altered antigen processing and presentation is a common feature of any pathogenic mycobacterial species and might not explain the characteristics of intestinal MAP infection (Kuehnel et al., 2001; Hostetter et al., 2002; Vergne et al., 2004). However, MAP additionally inhibits T cell and Dendritic cell (DC) activation and restricts the macrophage inflammatory cytokine response in cell culture systems (zur Lage et al., 2003; Basler et al., 2010, 2013). Thus, it seems that MAP persists and survives in its intestinal niche by subverting the immune defense mechanisms of the host and therefore might remain locally restricted to the intestine for long time (Atreya et al., 2014). In addition, on the bacterial side consequences of infection are characterized by a strong metabolic adaptation to the intestinal environment of the host (Weigoldt et al., 2011, 2013), which suggest that this capacity considerably adds to MAP pathobiology.

Within the MAC, MAP exhibits particular phenotypic features in culture. Thus, MAP growth in culture is dependent on mycobactin supplementation, extremely slow with the average doubling time of 22–26 h (other MAC ssp. 10–12 h), and shows a strong tendency to clump formation (Merkal and Curran, 1974). Differences in the phenotype and virulence of MAP might be linked to MAP specific genes (Li et al., 2005) and/or the acquisition, loss and rearrangement of specific genetic elements (Alexander et al., 2009). Sixteen large sequence polymorphisms (LSPs) were exclusively found in MAP. Six out of these 16 LSPs are common in all MAP strains and considered to be genomic insertions. These insertions comprise ~125 kb of DNA with 82 open reading frames (ORFs), with most of them being not of mycobacterial origin but exhibiting similarities to genes from environmental actinomycetes (Marri et al., 2006). Yet it is not clear whether the genes encoded on MAP-specific LSPs contribute to the phenotype and pathogenicity of MAP.

Previously, we had identified a MAP-specific putative 38 kb pathogenicity island which is located on LSP14, the largest LSP found in MAP. LSP14 harbors mostly genes which encode for proteins with predicted functions in metal metabolism (Stratmann et al., 2004). Within the 38 kb DNA region, a predicted operon *mpt*ABCDEF encodes for two putative ATP binding cassette transporters. The *mpt*ABCDEF operon (MAP3736c-31c) exhibits the highest similarity to other genera of the order Actinomycetales such as *Salinispora* or *Bifidobacter* and to a lesser extent to other mycobacteria. In addition, these genes have no other orthologs in MAP (data not shown). Among the operon genes, *mpt*D is unique in MAP within the genus of *Mycobacterium* since it is present in all sequenced cattle- and sheep-type MAP strains, but it is not found in any other so far sequenced mycobacterial species, even not in other subspecies of the MAC. Interestingly, *mpt*D was found to be surface-exposed and expressed during infection (Stratmann et al., 2004; Shin et al., 2006). In addition, Cossu and colleagues found antibodies against *mpt*D in sera of type I diabetes mellitus patients (Cossu et al., 2011), suggesting a possible role of *mpt*D in MAP pathogenicity in humans.

In the present study, we used specialized transduction to generate a *mpt*D knockout strain. By different comparative analyses we found that the gene is important for the metabolic homeostasis of MAP, which appears to be necessary for adaptation to the macrophage environment and survival in a mouse infection model.

## Materials and methods

### Bacterial strains, chemicals, and growth conditions

All chemicals were purchased from Sigma-Aldrich (Munich, Germany) if not stated otherwise. All bacterial strains, phages, and plasmids are listed in Table S1. The *Escherichia coli* strains DH5α and HB101 and the *M. smegmatis* mc^2^ 155 strain were cultured in Luria-Bertani (LB) broth or on LB agar plates (Roth, Karlsruhe, Germany) containing appropriate antibiotics with a concentration of 100 μg/ml. The *E. coli* strains DH5α and HB101 were used for cloning of homologous regions and for construction of the allelic exchange substrate (AES) in pYUB854. Additionally, *E. coli* HB101 was used for the *in vitro* λ-packaging reaction (GIGApack® II plus kit, Stratagene, La Jolla, CA, USA). For transformation experiments, competent *E. coli* HB101 were prepared following standard procedures (Sambrook and Russell, 2001). Liquid cultures of *E. coli* and *M. smegmatis* were grown at 37°C in a shaking incubator. *M. smegmatis* mc^2^ 155 was used for the generation of high titer phage lysates and phage construction as described (Carriere et al., 1997). *M. avium* subsp. *paratuberculosis* strain DSM 44135 (MAPwt) (Stratmann et al., 2004) was grown in Middlebrook 7H9 broth (MB7H9) or on Middlebrook 7H10 agar supplemented with 10% OADC and mycobactin J (2 mg/l; Allied Monitor, Fayette, USA). MB7H9 medium was further supplemented with Tween® 80 (0.05% final concentration) unless stated otherwise. For growth experiments, MAPwt and the mutant strain MAPΔ*mpt*D were cultured in supplemented MB7H9 medium until an OD_600_ of 3.0 was reached. The bacteria were then harvested, washed two times with phosphate-buffered saline (PBS) and diluted in supplemented MB7H9 without Tween® 80 to an OD_600_ of 0.2. Bacteria were cultivated with stirring at 120 rpm at 37°C and OD_600_ was measured every 2–3 days until entry of stationary phase.

### DNA techniques

Chromosomal mycobacterial DNA was prepared according to standard procedures (Belisle and Sonnenberg, 1998). Plasmids of *E. coli* were isolated using the NucleoSpin Plasmid kit (Macherey-Nagel, Düren, Germany) according to the manufacture's protocol. Southern blot analyses were performed with *Eco*RV-restricted chromosomal DNA according to standard protocols (Sambrook and Russell, 2001), using a α^32^-P-dCTP labeled PCR fragment obtained with primers omptD3a and omptD4a as a DNA probe. After hybridization, membranes were exposed to X-ray film (Kodak X-OMAT® Biomax, Sigma Aldrich GmbH, Deisenhofen, Germany). Polymerase-chain-reactions (PCR) were run on a Mastercycler system (Eppendorf, Hamburg, Germany), using HotStart HiFidelity Polymerase (Qiagen, Hilden, Germany) and the following conditions: 95°C for 10 min, 95°C for 45 s, 58°C for 60 s, and 72°C for 60 s. The oligonucleotides are listed in Table S1.

### Construction of MAPΔ*mpt*D

**Figure 1:**
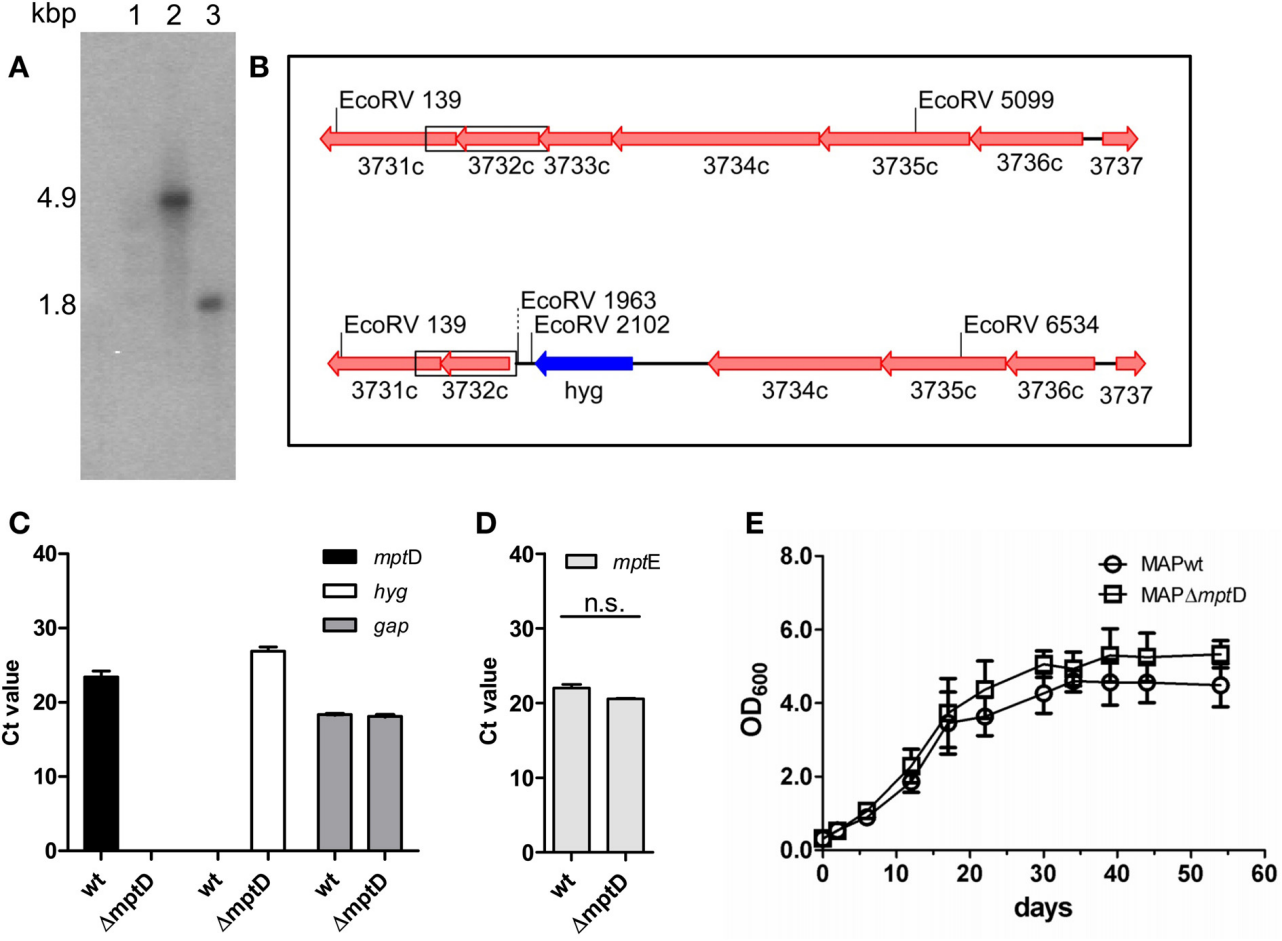
**Confirmation and characterization of MAPΔ*mpt*D mutant by Southern blot analysis (A,B), qRT-PCR (C,D) and growth experiments (E). (A)** EcoRV restricted genomic DNA of MAPwt (lane 2) and MAPΔ*mpt*D mutant strain (lane 3), Southern blotted and hybridized with a probe against *mpt*E (MAP3732c). In the wild type a 4.9 kbp fragment is labeled, the mutant strain shows a 1.8 kbp fragment. Lane 1 DNA marker. **(B)** Genomic sketch of the *mpt* region spanning genes *mpt*F (MAP3731c) to *mpt*A (MAP3736c) in MAPwt (top) and Δ*mpt*D mutant strains (bottom). Position of EcoRV restriction sites are indicated, the α^32^-P-dCTP labeled probe fragment is boxed. **(C)** Transcription of *mpt*D (black bars) and hygromycin (hyg) (white bars) in MAP wild type (wt) and MAPΔ*mpt*D were analyzed by qRT-PCR. As a control, cDNA samples were tested for the housekeeping gene *gap* (gray bars). **(D)** Analysis of *mpt*E (MAP3732c) transcription in MAPwt and MAPΔ*mpt*D by qRT-PCR (n.s., no significant difference). **(E)** Growth of MAPwt and MAPΔ*mpt*D in Middlebrook 7H9 medium. Cultures were inoculated with an initial OD_600_ of 0.2 and growth was monitored at OD_600_ (ordinates) at different time points (abscissa) until stationary phase. The results of **(C,D)** represent the mean ± standard error (s.e.m.) of three replicates.

The construction of the specialized transducing mycobacteriophage containing the AES was performed according to Park and colleagues (Park et al., 2008) with slight modifications. A detailed protocol is given in the supplementary methods file. Briefly, in order to generate an *mpt*D (MAP3733c) deletion in MAP, two oligonucleotide primer pairs (Table S1) were generated to amplify the up- and downstream flanking regions of the *mpt*D gene resulting in an deletion of 518 bps of the *mpt*D ORF upon restriction digest and ligation of the amplified products into the cosmid vector pYUB854 (Bardarov et al., 2002). The resulting construct pMP1310 was introduced into the mycobacterial phage vector phAE87 (Bardarov et al., 1997), resulting in phage phAE111. To construct a MAPΔ*mpt*D mutant, MAP was cultured in MB7H9 medium to OD_600_ of 1.0. Bacterial suspensions of MAP, free of clumps, were transduced and plated on MB7H10 agar plates containing 50 μg/ml hygromycin B and incubated at 37°C for up to 12 weeks. Successful recombination was confirmed by PCR, Southern blot and quantitative real-time PCR (qRT-PCR) analyses (Figures 1A–C).

### PacBio *RSII* resequencing study

SMRTbell™ template library was prepared according to the instructions from PacificBiosciences, Menlo Park, CA, USA, following the Procedure and Checklist Greater than 10 kb Template Preparation and Sequencing. Briefly, for preparation of 10 kb libraries, ~10 μg genomic DNA were end-repaired and ligated overnight to hairpin adapters applying components from the DNA/Polymerase Binding Kit P4 from Pacific Biosciences, Menlo Park, CA, USA. Reactions were carried out according to the manufacturer's instructions. SMRTbell™ template was Exonuclease treated for removal of incompletely formed reaction products. Conditions for annealing of sequencing primers and binding of polymerase to purified SMRTbell™ template were assessed with the Calculator in RS Remote, PacificBiosciences, Menlo Park, CA, USA. SMRT sequencing was carried out on the PacBio *RSII* (PacificBiosciences, Menlo Park, CA, USA) taking one 180-min movie for each SMRT cell. In total 1 SMRT cell was run.

### RNA extraction and quantitative real-time PCR

Total RNA was extracted from MAPwt and MAPΔ*mpt*D grown to mid log phase (OD_600_ of 1.0) using a TRIzol®protocol according to Rustad et al. (2009). RNA was additionally purified using an RNeasyMini kit (Qiagen, Düsseldorf, Germany) with DNase I (50 U) in tube treatment according to the manufacturer's manual. DNAse digested RNA was used for double strand cDNA-synthesis. Briefly, in a total volume of 20 μl, 4 μg of RNA were mixed with 0.4 μg random primers (Promega, Madison, WI, USA), incubated for 10 min at 70°C in a thermal cycler and subsequently cooled on ice for 5 min. Aliquots (10 μl) were mixed with 5 μl 5× reaction buffer and 2 μl 10 mM dNTP's in a total volume of 25 μl. Reverse transcription was performed by adding either 200 U MMLV-superscript transcriptase (Promega, Madison, WI, USA) or RNase free water as a negative control. Reactions were incubated for 1 h at 42°C, followed by an incubation for 5 min at 85°C. Samples were diluted with 90 μl ddH_2_O and stored at −20°C until further analysis.

For real-time PCR experiments, 2.5 μl of cDNA were mixed with 0.4 μM primer each and 10 μl SYBR-Green Mix (Qiagen, Hilden, Germany) in a total volume of 20 μl and subsequently analyzed using a Mx3005P qPCR system (Stratagene, Agilent Technologies, La Jolla, CA, USA) with a thermal cycling profile as follows: segment 1, 20 min at 95°C, 1 cycle; segment 2, 45 s at 95°C, 1 min at 58°C, 1 min at 72°C, 45 cycles; segment 3, 1 min at 95°C, 30 s at 55°C, 30 s at 95°C, 1 cycle. Results were normalized to the housekeeping gene *gap* (MAP1164) and expressed as fold-change to the untreated control.

### Metabolomic screen

For metabolomic screening, three independent biological replicates of MAPwt and MAPΔ*mpt*D were grown to mid log phase (OD_600_ of 1.0) in supplemented MB7H9 medium. To stop metabolic activity, 15 ml of each culture were transferred to a quenching solution (60% methanol kept at −20°C) in a dilution of 1:3 (vol/vol). Bacteria were harvested by centrifugation at 4°C at 8000 × g for 3 min, pellets were washed with 1 ml quenching solution and bacteria were pelleted again. Supernatants were removed and pellets were resuspended in 1 ml 80% methanol (−20°C) for metabolite extraction. Ten microliter of an internal standard (0.2 mg of ^13^C6 labeled sorbitol in 1 ml methanol) were added to the samples. Samples were transferred to a Fastprep tube and disrupted in a bead beater using four cycles at intensity setting 6.5 for 40 s with intermediate cooling on ice. Samples were incubated at 70°C for 15 min, centrifuged at 4°C at 4000 × g for 15 min, and 10 μl were transferred to an appropriate glass tube for freeze-drying overnight. Dried samples and 300 μl of undried samples were used for gas and liquid chromatography (GC/LC) analyses, respectively. All subsequent steps were carried out at Metabolomic Discoveries GmbH (Potsdam, Germany; www.metabolomicdiscoveries.com). Derivatization and analysis of metabolites in a GC-MS 7890A mass spectrometer (Agilent, Santa Clara, USA) were carried out as described (Lisec et al., 2006). Metabolites were identified in comparison to Metabolomic Discoveries' database entries of authentic standards. The LC separation was performed using hydrophilic interaction chromatography with a ZIC-HILIC 3.5 μm, 200 A column (Merck Sequant, Umeå, Sweden), operated by an Agilent 1290 UPLC system (Agilent, Santa Clara, CA, USA). The LC mobile phase was a linear gradient from 90 to 70% acetonitrile over 15 min, followed by a linear gradient from 70 to 10% acetonitrile over 1 min, 3 min wash with 10% and 3 min reequilibration with 90% acetonitrile. The flow rate was 400 μl/min, the injection volume was 1 μl. The mass spectrometry was performed using a 6540 QTOF/MS Detector (Agilent, Santa Clara, CA, USA). The measured metabolite concentration was normalized to the internal standard. Significant concentration changes of metabolites in different samples were analyzed by normal distribution (Shapiro–Wilk-Test) and variance homogeneity testing (*F*-Test) using appropriate statistical test procedures (Students test, Welch test, Mann–Whitney test) (see Tables S2A,B). A *p*-value of *p* < 0.05 was considered as significant.

### Lipid profiling

MAPwt and MAPΔ*mpt*D (400 and 600 mg lyophilized cells, respectively) were suspended in 20 ml of a mixture of chloroform/methanol (2:1, v/v) and extracted by sonication on ice (Branson sonifier, output 4, duty cycle 40, 2 × 15 min, pulsed). After centrifugation at 3000 × *g* for 30 min at 4°C, the supernatant was transferred and dried using a rotary evaporator yielding 100 and 170 mg of crude lipids, respectively. The crude lipids were fractionated according to their polarity on 10 × 1.5 cm columns filled with Silica gel K60 (0.04–0.063 mm mesh size, Merck, Darmstadt, Germany) sequentially using chloroform, acetone and methanol (100 ml each), followed by a final wash with 10 ml of a mix containing equal volumes of chloroform and methanol.

All fractions were evaporated and dry masses determined. The lipid fractions were analyzed on high-performance thin-layer chromatography (HPTLC) plates (Merck, Darmstadt, Germany). Briefly, 10 μg of each lipid fraction were applied and resolved either in chloroform/methanol (9:1) or chloroform/methanol/water (65:25:4). Commercial trehalose dimycolate (TDM; Bioclot GmbH, Aidenbach, Germany), and a mixture of phosphoinositol mannosides (PIMs) extracted from *M. tuberculosis* were included as marker. After evaporation of the solvent, lipid bands were visualized by dipping the plates in a solution of Hanessian's stain [2.5 mM cerium (IV) sulfate, 40 mM ammonium hepta-molybdate in 5.8% H_2_SO_4_] followed by heating to 150°C for 5 min.

### Macrophage cell culture and viability assessment of intracellular mycobacteria

The mouse macrophage cell lines J774A.1 (Ralph et al., 1975) and RAW264.7 (Raschke et al., 1978) were maintained in Dulbecco's modified Eagle medium (DMEM) supplemented with 10% FCS, 1% glutamine, 100 units/ml penicillin, 100 μg/ml streptomycin at 37°C and 8% CO_2_. For infection experiments, cells were maintained in antibiotic-free DMEM for 48 h. To determine mycobacterial viability during infections, macrophages were infected as described (Kuehnel et al., 2001) with MAPwt and MAPΔ*mpt*D in a multiplicity of infection (MOI) of 10:1. After infection at the indicated time points, monolayers were washed twice with PBS and scraped off the plates in 1 ml of 1% Nonidet P40 in PBS. Cells were disrupted by 10 passages through a 24-gauge needle. A 10-fold serial dilution of the homogenates was prepared in PBS and 100 μl of each dilution level were plated on supplemented Middlebrook 7H10 agar plates. After incubation for up to 12 weeks at 37°C, the colony-forming units (cfu) were counted.

### Bacterial adhesion assay and flow cytometry

Adhesion of mycobacteria was assayed by flow cytometry as described previously (Pott et al., 2009; Basler et al., 2010). Briefly, mycobacteria grown to an OD_600_ of 1.0 were fluorescently labeled using carboxyfluorescein succinimidyl ester (CFSE, Life Technologies GmbH, Darmstadt, Germany) at 10 μM final concentration in PBS for 20 min at 37°C. After two washes in PBS, labeled bacteria were resuspended in DMEM and used for infection of macrophages. To quantify mycobacterial adhesion to J774.A1 macrophages, confluent grown cells were pretreated with latrunculin (1 μg/ml) for 30 min to inhibit phagocytosis and subsequently incubated for 1 h with CFSE labeled MAPwt and MAP Δ*mpt*D in a MOI of 10:1. Then, bacteria-containing medium was removed, and cells were scraped off in PBS, washed for 5 min with PBS by overhead shaking at 4°C, and pelleted by centrifugation at 250 × g for 5 min. Washing was repeated twice. Cells were analyzed with a FACSCalibur (BD biosciences, San Jose, CA, USA). Results were expressed as means ± standard error of the mean (SEM) fluorescence intensities.

### Animal experiment

The mouse infection experiments were approved by the Lower Saxony Federal State Office for Consumer Protection and Food Safety, Germany (reference number 08/1504). Female C57BL/6 mice aged 8 weeks (Charles River, Erkrath, Germany) were used. Mice were infected intraperitonially (i.p.) with an infection dose of 1 × 10^8^ or 2 × 10^8^ pathogens of each strain in 200 μl Dulbecco's Phosphate-Buffered Saline (DPBS, Life Technologies GmbH, Darmstadt, Germany; 10 mice per group). Application of PBS was used as negative control. Two days postinfection the body mass monitoring started and was repeated three times per week. After 4 weeks mice were sacrificed, liver, spleen and mesentery tissue were weighted, and MAP (MAPwt and MAPΔ*mpt*D) were quantified by plating of the homogenized tissue on MB7H10 agar plates supplemented with 10% OADC and mycobactin.

Histology was performed in the Mouse Pathology platform at HZI Braunschweig. Briefly, organs were fixed in 10% formaldehyde (v/v), dehydrated with ethanol, and embedded in paraffin. Paraffin sections (0.5 μm) were stained with hematoxylin-eosin (HE) according to standard laboratory procedures. HE stained slices of liver were investigated at 400 × magnification and granuloma counts per area were determined manually using AxioVision Le software (Carl Zeiss AG, Jena, Germany) based on the contrast between granulomas (blue) and other cells of the liver (pink). To minimize false positive results, only granuloma with a size of >2000 μm^2^ were counted and compared to the liver slices of uninfected mice, where no granuloma have been detected.

### Statistics

Data are expressed as mean ± s.e.m. By using GraphPad Prism 4.0 (GraphPad, San Diego, CA, USA) a parametric *t*-test or One-Way ANOVA tests were used for statistical analyses of macrophage and the animal experiment. Differences between treated samples and controls were considered statistically significant at a level of *p* < 0.05.

## Results

### Construction of MAPΔ*mpt*D

In order to gain insight into the role of *mpt*D in MAP pathogenicity we generated a Δ*mpt*D mutant strain by specialized transduction (Braunstein et al., 2002; Park et al., 2008). For this, the *mpt*D gene (MAP3733c) in MAP strain DSM44135 was replaced by a hygromycin cassette. Hyg-resistant colonies were picked and deletion of *mpt*D was confirmed by Southern blot hybridization and qRT-PCR analyses (Figures 1A–C). A positive clone designated MAPΔ*mpt*D was further propagated. To exclude polar effects by the mutation on downstream genes, RNA extracted from OD_600_ of 1.0 cultures of MAPΔ*mpt*D and MAPwt was analyzed by qRT-PCR. As shown in Figure 1D, the mRNA expression level of the downstream gene *mpt*E (MAP3732c) was not affected in MAPΔ*mpt*D, excluding polar effects. On the other hand, this implied that the following genes *mpt*E and *mpt*F were not part of the predicted *mpt*ABCDEF.

Any illegitimate recombination in MAPΔ*mpt*D could be excluded by PacBio resequencing study applying the RefSeq genome of strain MAP-K10 (NC_002944.1, Li et al., 2005) in the RS_Resequencing.1 protocol of SMRTPortal 2.1.1. Hereby, coverage analysis using samtools mpileup (PMID 19505943) on the resulting BAM-file confirmed no further large-scale deletions in MAPΔ*mpt*D. Alignment analyses of PacBio long reads by BLAST confirmed that the hygromycin cassette was inserted nonrecurring and at exactly that position as expected.

Following, we analyzed growth of MAPΔ*mpt*D and MAPwt in supplemented MB7H9 broth (Figure 1E). Both wild type and mutant strains grew with similar kinetics and entered stationary phase after 34 days. This indicated that *mpt*D is not necessary for growth of MAP in MB7H9.

### Reduced intracellular survival of MAPΔ*mpt*D in macrophages

**Figure 2:**
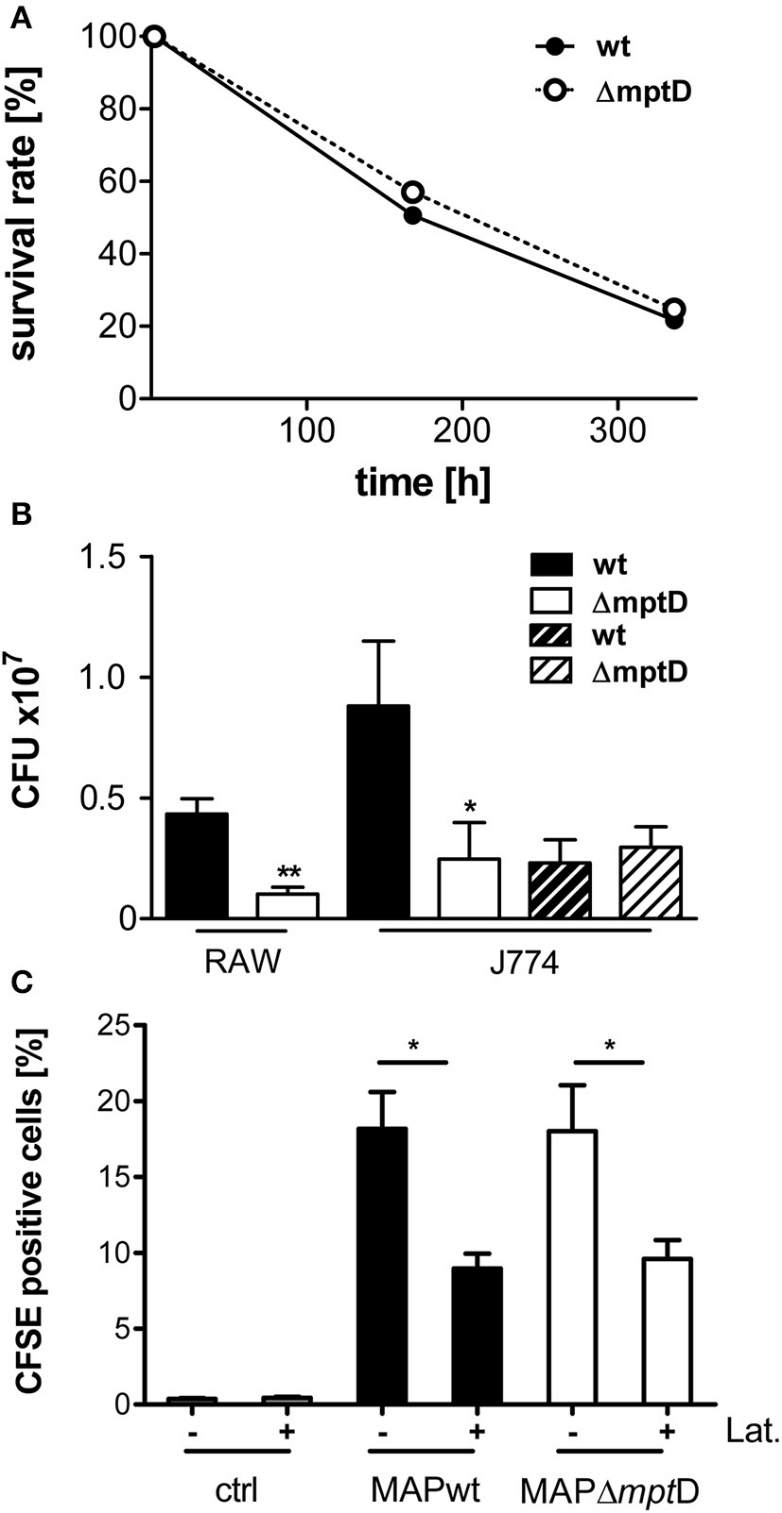
**Survival and association of MAPwt and MAPΔ*mpt*D strain in mouse macrophages. (A)** Survival rates of MAPwt and MAPΔ*mpt*D in RAW264.7 macrophages. Exponentially growing bacteria of MAPΔ*mpt*D and MAPwt were harvested at OD_600_ of 1.0, and single cell suspensions of an OD_600_ of 0.1 in DMEM were used to infect murine RAW264.7 macrophages as described in Materials and Methods. CFU were counted at 2 h, 8 and 14 days of macrophage infection. Intracellular survival rates were calculated by relating the CFU after 8 and 14 days to those of 2 h. **(B)** CFU numbers of bacteria after infection of mouse macrophages (RAW264.7/J774.A1) with MAPwt and MAPΔ*mpt*D for 2 h (filled bars) and after incubation of the strains in macrophage cell lysates for 2 h (dashed bars). The results represent the mean ± standard error (s.e.m.) of at least three independent replicates for the macrophage infections and two replicates for the incubation in macrophage lysates. **(C)** Association and invasion of CFSE labeled MAP wild type and MAPΔ*mpt*D strains in J774.A1 macrophages after 2 h incubation with (+) and without (−) latrunculin (Lat.). The statistical analysis was performed using a parametric *t*-test (CFU) or a One-Way ANOVA combined with Dunnett's multiple comparison test (CFSE experiments). A *p*-value ≤ 0.05 was defined as statistically significant (^**^p < 0.005; ^*^p < 0.05).

To investigate the potential role of *mpt*D in pathogenicity, we performed survival experiments in murine RAW264.7 macrophages and also included J774A.1 macrophages of which we had detailed information of the phagosomal acidification process (Kuehnel et al., 2001). Exponentially grown bacteria of MAPΔ*mpt*D and MAPwt were harvested at an OD_600_ of 1.0, and single cell suspensions of an OD_600_ of 0.1 in DMEM were used to infect macrophages as described in Materials and Methods. CFU were counted at 2 h, 8 and 14 days of macrophage infection. Intracellular survival rates were calculated by relating the CFU after 8 and 14 days to those at 2 h. By this, we observed similar intracellular survival rates for MAPwt and MAPΔ*mpt*D representatively shown for RAW264.7 macrophages in Figure 2A. An interesting observation of these experiments was, however, that the number of cultivable MAPΔ*mpt*D after 2 h of infection was significantly lower as compared to MAPwt (Figure 2B, filled bars). This was true for MAP infection of RAW264.7 and J774A.1 macrophages (Figure 2B). However, incubating MAPwt and MAPΔ*mpt*D in macrophage whole cell lysates for 2 h led to similar bacterial CFU counts indicating that exponentially growing mutant and wild type strain had similar capability to survive in this environment (Figure 2B, dashed bars).

Next we analyzed whether the lower levels of cultivable MAPΔ*mpt*D after macrophage infection resulted from lower adhesion of MAPΔ*mpt*D to macrophages and/or decreased up-take of MAPΔ*mpt*D by the macrophages. For this, we performed FACS analyses of untreated and latrunculin-treated J774A.1 macrophages incubated with CFSE-labeled MAPwt and MAPΔ*mpt*D. Non-infected macrophages were included as negative control. As shown in Figure 2C, no difference between MAPwt and MAPΔ*mpt*D bacteria at the level of general association with macrophages (adherent and intracellular mycobacteria) was observed. Also the level of adhesion was similar (Figure 2C, latrunculin treated macrophages). Together these results suggested that the reduced cell number of MAPΔ*mpt*D in macrophage compartments was due to a hampered adaptation of MAPΔ*mpt*D to the early phagosomal environment of macrophages.

### MAPΔ*mpt*D is attenuated after peritoneal challenge of mice

**Figure 3:**
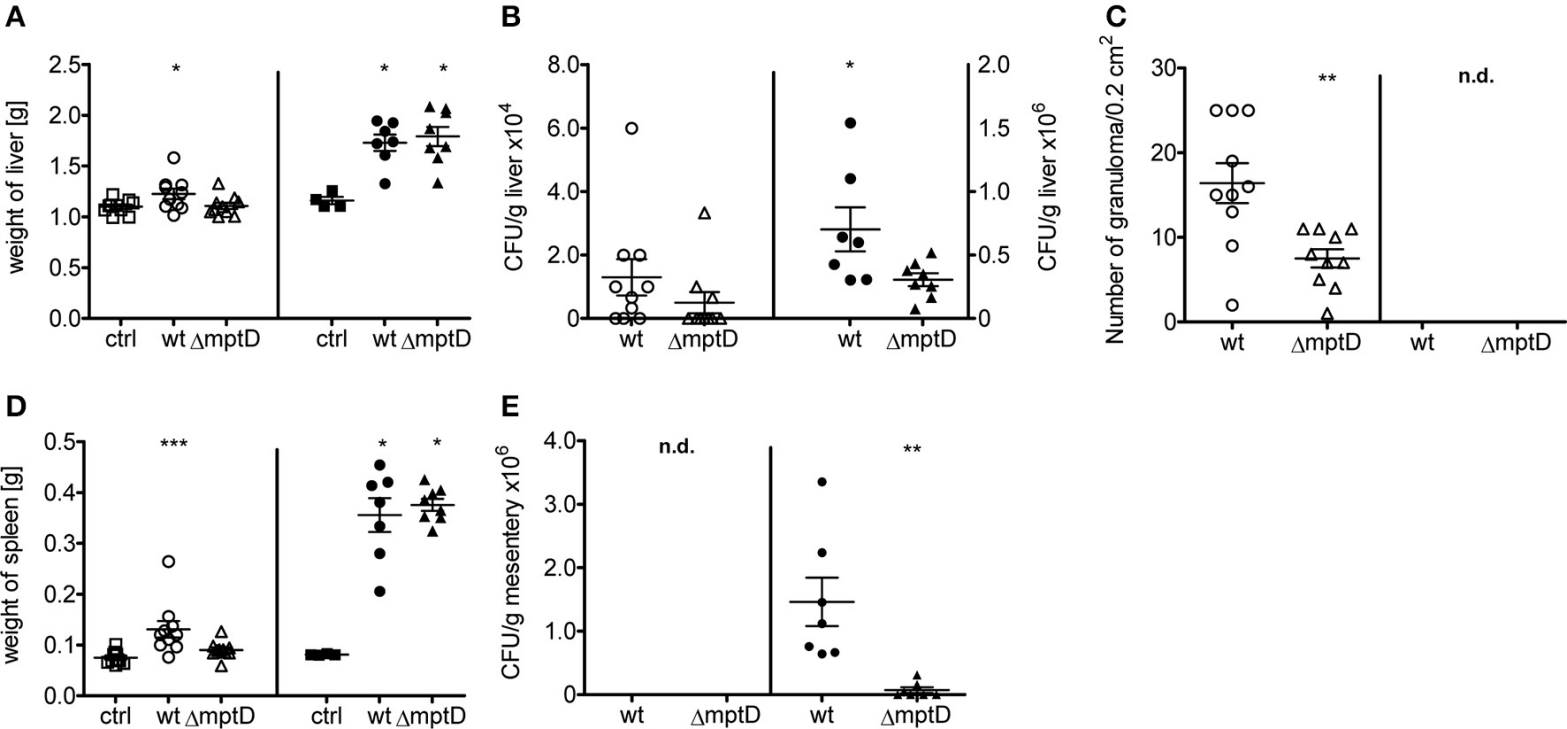
**Biological fitness of MAP wild type (MAPwt) and Δ*mpt*D mutant strain (MAPΔ*mpt*D) in infected mice**. C57BL/6 8 week old female mice were infected with either 1^*^10^8^ (open symbols) or 2^*^10^8^ cells (black symbols) of MAPwt (circle) and MAPΔ*mpt*D (triangle) for 4 weeks. DPBS buffer was used for the control group (ctrl, squares). The read out parameters for this experiment were the weight of liver **(A)** and spleen **(D)**, CFU detection of bacteria from liver **(B)** and mesentery **(E)** as well as the amount of granuloma in the liver **(C)**. The results represent the mean ± standard error (s.e.m.) of animal experiments with 7–10 mice of each group. The statistical analysis was performed using One-Way ANOVA analysis (Kruskal–Wallis test). A *p*-value ≤ 0.05 was defined as statistically significant (^***^p < 0.0005; ^**^p < 0.005; ^*^p < 0.05).

The infection experiments of macrophage cultures revealed that MAPΔ*mpt*D is hampered in intracellular survival and indicated that *mpt*D might be important for survival in the host. Therefore, to analyze survival of MAPΔ*mpt*D and MAPwt in a more complex system, we infected C57BL/6 mice intraperitoneally with 1 × 10^8^ or 2 × 10^8^ bacteria of exponentially growing cultures of OD_600_ of 1.0 and animals were sacrified 4 weeks post infection. As shown in Figure 3, compared to the untreated controls, mycobacteria infected mice exhibited increased liver and spleen weight which was more pronounced in the animals infected with the higher infection dose (Figures 3A,D). In lower dose infections, a significant increase in spleen weight was observed in wild type-infected mice but not in mice infected with the mutant (Figure 3D). The CFUs in liver tissue were higher in wild type-infected than in mutant-infected mice (Figure 3B). These differences became significant when the higher infection dose was used. Correspondingly, a higher number of granuloma was counted in HE-stained histological sections (Figure 3C, Suppl. Figure S1), however, no significant difference in granuloma sizes was detected (data not shown). We have recently observed that after i.p. infection of mice, MAP persists in large numbers in mesenteric macrophages (Suwandi et al., in press). Therefore, we also analyzed CFU in the mesentery. As shown in Figure 3E, also in this tissue MAPΔ*mpt*D was isolated in significantly lower numbers than MAPwt. Overall, our animal experiments indicate a significantly lower biological fitness of MAPΔ*mpt*D in the mouse infection model.

### MAPΔ*mpt*D exhibits an altered lipid metabolism

**Figure 4:**
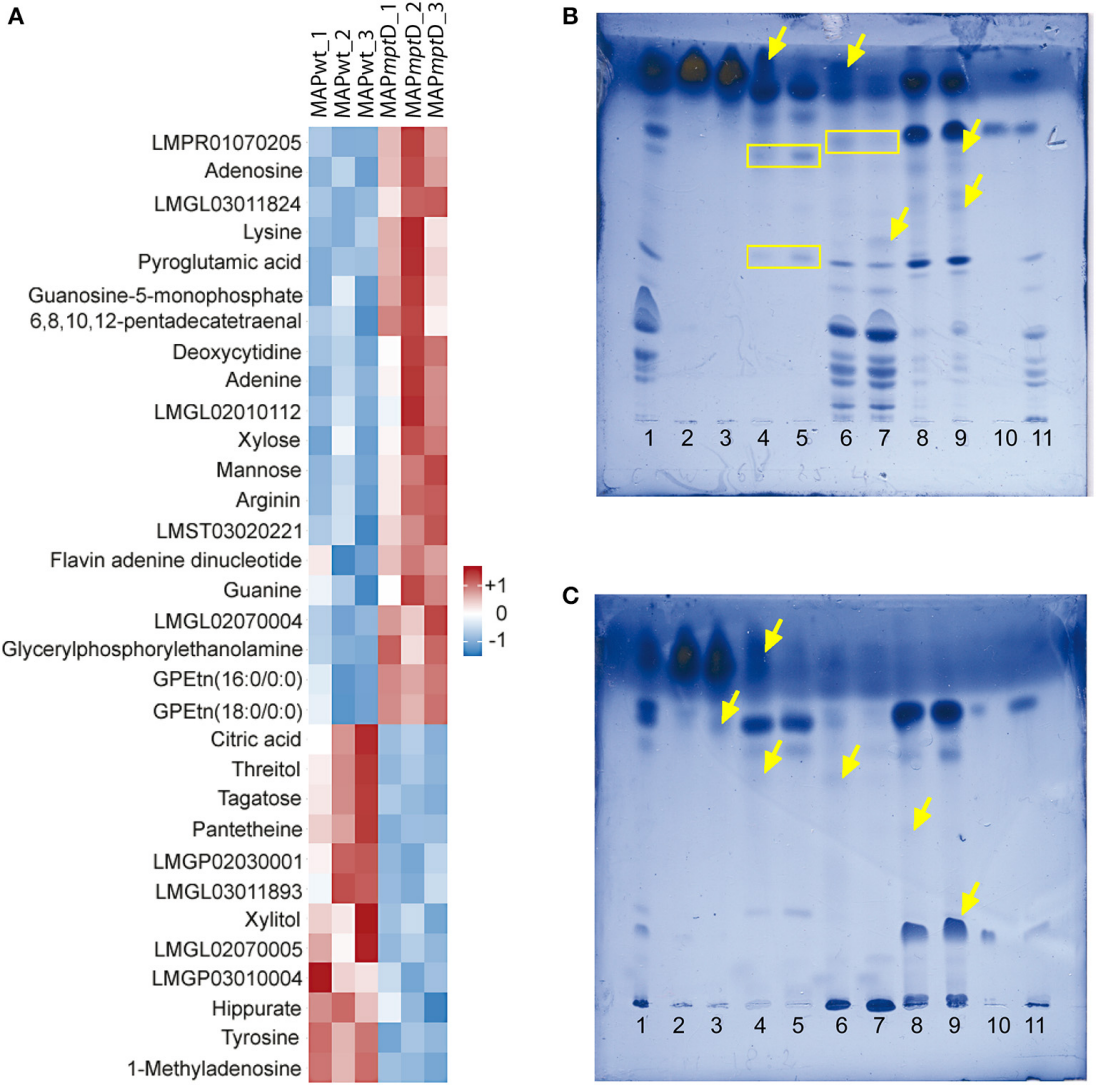
**GC/LC-MS analysis of metabolites (A) and HPTLC analysis of lipids (B,C) from MAPwt and MAPΔ*mpt*D. (A)** The heat map illustrates the significant concentration changes (overall results of three biological replicates). The color intensity and tones represent magnitude and direction of metabolic changes, respectively, with the magnitude of change ranging from white (reference) to red (positive deviation to reference) or blue (negative deviation to reference). **(B,C)** HPTLC analysis of polarity-fractionated lipids. Mobile phase in **(B)** chloroform/methanol 18:2 (v/v); in **(C)** chloroform/methanol/water 65:25:4 (v/v/v). Lane 1: crude lipids from MAPΔ*mpt*D; lanes 2, 4, 6 and 8: fractions of MAPwt; lanes 3, 5, 7, and 9: fractions of MAPΔ*mpt*D; lanes 2 and 3: chloroform fraction; lanes 4 and 5: acetone fraction; lanes 6 and 7: methanol fraction; lanes 8 and 9: chloroform/methanol 1:1 (v/v) column wash; lane 10: TDM standard; Lane 11: PIM standard (see text) yellow arrows = additional bands; yellow boxes = same bands with different intensities.

In order to determine the functional cause for the decreased survival of MAPΔ*mpt*D in macrophages and its attenuation in a mouse infection model, comparative metabolomic profiling was performed to detect differences in metabolite concentrations between MAPΔ*mpt*D and its parental strain. The concentrations of 175 metabolites in MAPwt and the MAPΔ*mpt*D were compared (Suppl. Table S2). Among the 175 metabolites, the abundances of 32 were significantly different (Figure 4A). Means and standard deviation for the three experiments are shown in Table 1. Different relative abundances for 13 lipids and lipid-intermediates, 5 carbohydrates (mannose, xylose, xylite, tagatose, threitol), 4 amino acids (lysine, arginine, pyroglutamine, tyrosine), 2 nucleobases (guanine, adenine), 4 nucleotides (adenosine, deoxycytidine, guanosine-5-phosphate, methyladenosine), as well as the abundance of hippurate, citric acid, FAD and the CoA precursor panthetheine were found in MAPΔ*mpt*D. Twelve metabolites had a reduced abundance and 20 an increased abundance in MAPΔ*mpt*D (Table 1).

**Table 1:** **Relative abundance of significantly differential metabolites (MAPΔ*mpt*D vs. MAPwt)**.

**Metabolite name**	**Technique**	**Fold change**	**Metabolic classification**
LMGL03011824^*^	LC	11.8	Lipid transport and metabolism
Lysine	GC	8.0	Amino acid metabolism
Glycerylphosphorylethanolamine	LC	7.1	Lipid transport and metabolism
LMPR01070205^*^	LC	6.5	Lipid transport and metabolism
LMGL02010112^*^	LC	5.0	Lipid transport and metabolism
Deoxycytidine	LC	4.2	Nucleic acid metabolism
LMST03020221^*^	LC	3.6	Lipid transport and metabolism
LMFA06000087^*^	LC	3.1	Lipid transport and metabolism
Adenosine	LC	3.0	Nucleic acid metabolism
Arginine	LC	2.8	Amino acid metabolism
GPEtn(18:0/0:0)	LC	2.7	Lipid transport and metabolism
Pyroglutamic acid	GC	2.3	Amino acid metabolism
Adenine	LC	2.1	Nucleic acid metabolism
Guanosine-5-monophosphate	GC	2.1	Nucleic acid metabolism
LMGL02070004^*^	LC	2.0	Lipid transport and metabolism
GPEtn(16:0/0:0)	LC	1.9	Lipid transport and metabolism
Guanine	LC	1.8	Nucleic acid metabolism
Mannose	GC	1.7	Carbohydrate metabolism
Xylose	GC	1.7	Carbohydrate metabolism
Flavin adenine dinucleotide (FAD)	LC	1.4	Co-factor metabolism
LMGL02070005^*^	LC	−1.4	Lipid transport and metabolism
Hippuric acid	LC	−1.5	Amino acid metabolism
Xylitol	GC	−1.6	Carbohydrate metabolism
LMGP02030001^*^	LC	−1.6	Lipid transport and metabolism
Tyrosine	GC	−2.0	Amino acid metabolism
LMGP03010004^*^	LC	−2.4	Lipid transport and metabolism
1-Methyladenosine	LC	−2.5	Nucleic acid metabolism
Citric acid	GC	−2.7	Carbohydrate metabolism
Threitol	GC	−3.1	Carbohydrate metabolism
Tagatose	GC	−3.6	Carbohydrate metabolism
Pantetheine	LC	−7.6	Co-factor metabolism
LMGL03011893^*^	LC	−7.7	Lipid transport and metabolism

* LIPID MAPS ID according to the LIPID MAPS Structure Database (http://www.lipidmaps.org/data/structure/index.html).

To examine the consequences of the marked differences in lipid metabolites, crude lipids were extracted from MAPwt and MAPΔ*mpt*D, fractionated on silica gel columns and analyzed by thin-layer chromatography. In almost all fractions, we found differential band pattern of lipids in MAPΔ*mpt*D compared to its parental strain (Figures 4B,C). These analyses confirmed the severe changes in lipid metabolism suggested by our metabolic analyses. Overall, these data clearly show the presence of a deregulated lipid metabolism with severe alteration of central metabolic processes in MAPΔ*mpt*D.

## Discussion

The pathogenesis of JD is still only partially resolved. In contrast to other pathogenic mycobacteria, MAP is very slow growing and mycobactin-dependent in culture (Merkal and Curran, 1974). Furthermore, unlike the related *M. avium* ssp. *hominissuis*, MAP exhibits a strong tissue tropism to the gut which is not seen in other mycobacteria. These phenotypical features might be attributed to MAP-specific genotypical features. Thus, MAP possesses eight common MAP-specific LSPs (Alexander et al., 2009). Thirty-four MAP genes are not present in other mycobacteria species; 13 of these genes are most probably acquired from other Actinomycetales and 21 genes without a homology to other bacteria have been sequenced so far. Although MAP-specific elements are promising candidates for explaining the peculiarities of MAP, the knowledge concerning their relevance in pathogenicity is poor.

The *mpt*D gene is one of the MAP-specific genes not present in any other yet sequenced mycobacterial species. It is expressed from the 38 kb pathogenicity island within the LSP14. LSP14 contains mainly genes assigning to metal acquisition (Stratmann et al., 2004; Alexander et al., 2009). This predicted function refers a particular importance to LSP14 as it might contain a putative, alternative iron uptake locus, supplementing for MAP mycobactin deficiency caused by a truncated *mbt*A gene (Li et al., 2005). In addition to its location on LSP14, the fact that *mpt*D was shown to be expressed during infection (Stratmann et al., 2004) suggests a possible role of *mpt*D in metabolism in the host environment and in pathogenicity.

Our studies with the Δ*mpt*D mutant strain clearly denote the important standing of *mpt*D for MAP metabolism and pathogenicity. We were not able to restore the MAPΔ*mpt*D phenotype by complementation of the MAPΔ*mpt*D with wild-type *mpt*D or the complete operon (data not shown), most likely due to the complex organization of the predicted transporter proteins and their yet unknown regulation. Yet we found no evidence for alterations on the genome level except the deletion of the gene itself which might be responsible for the MAPΔ*mpt*D phenotype.

Overall, MAPΔ*mpt*D was considerably hampered with respect to survival in macrophages and in mice. Most probably MAPΔ*mpt*D is not fully able to resist phagosomal factors to which it is exposed during the first 2 h after infection. This is strongly suggested since the logarithmically grown bacteria showed similar survival after 2 h of incubation in the macrophage cytosol but the survival of MAPΔ*mpt*D was strongly reduced after 2 h macrophage infection. The observed long-term survival of MAPΔ*mpt*D after infection of macrophages and in mice, however, indicates that the absence of *mpt*D does not lead to a general attenuation thereby indicating an important role of *mpt*D in early infection immediately after encountering the hostile environment of the early phagocytic vacuole.

Our metabolic analyses clearly show that reduced survival is correlated with an altered metabolism of MAPΔ*mpt*D. Metabolic analyses revealed a reduced activity of the TCA cycle in MAPΔ*mpt*D which was indicated by the negative deviation of citric acid. This might be caused by a lower availability of acetyl-CoA induced by an enhanced acetyl-CoA consuming fatty acid synthesis, which is suggested by the accumulation of nine fatty acid metabolites. In addition, the activity of the TCA cycle appears to be stressed by carbon efflux used for the generation of arginine and lysine. Increased fatty acid synthesis is also reflected by a higher activity of the pentose phosphate pathway which is deducible from the accumulation of nucleosides and FAD. The three fatty acid metabolites with negative deviation and the accumulation of FAD indicate an enhanced β-oxidation of fatty acids to generate acetyl-CoA in MAPΔ*mpt*D which, however, seems not to satisfy the demand. This metabolic derailment in MAPΔ*mpt*D might be enforced by a diminished CoA availability as indicated by significantly diminished levels of pantetheine, an intermediate for CoA formation. Furthermore differential carbohydrate conversion in MAPΔ*mpt*D is indicated by the accumulation of mannose and xylose, a precursor of arabinose (Wolucka, 2008). Mannose and arabinose are principal components of important cell envelope constituents such as lipoamannan (LM), lipoarabinomannan (LAM), and arabinogalactan (Brennan, 2003).

MAP possesses ~300 genes involved in lipid metabolism and the highest number of redundant genes for this metabolic extent among pathogenic mycobacteria (Li et al., 2005; Marri et al., 2006). The most obvious changes in MAPΔ*mpt*D were seen in the lipid metabolism (Figures 4B,C). This is in accordance with the reduced survival in macrophages and in mice since fatty acid metabolism in pathogenic mycobacteria has been shown to be essential for the survival of the bacterium in the host (Russell et al., 2010). On the one hand, the solid cell wall with mycolic acids and lipoglycans such as LAM and LM, produced from elongated fatty acids, provides a protective lipid layer (Rowe and Grant, 2006; Hett and Rubin, 2008), on the other hand lipids are implicated as major nutrient sources of pathogenic mycobacteria in the host. Thus, mRNA expression analysis of *M. tuberculosis* (Mtb) obtained from macrophages *in vitro* and from the lungs of mice and humans implied that Mtb changes its intermediary metabolism *in vivo* by using host-derived lipids such as cholesterol during the course of infection rather than using glucose and glycerol, the primary carbon sources metabolized *in vitro* (Schnappinger et al., 2003; Timm et al., 2003; Talaat et al., 2004). More recently, it was shown that cholesterol degradation appears to be important for feeding Mtb during chronic infection (Miner et al., 2009). Accordingly, we have recently shown that, in the host, MAP increases the activity of the TCA cycle by enhancing β-oxidation of lipids, most probably cholesterol (Weigoldt et al., 2013).

Our data indicate that a dysfunction of the balanced lipid metabolism necessary for survival of MAP in the host might be responsible for the attenuation of MAPΔ*mpt*D in macrophages and in mice. The exceptional role of the mycobacterial lipid metabolism for mycobacterial survival has been emphasized in many studies (Russell et al., 2010). Disorders in the lipid homeostasis can dramatically influence mycobacterial viability. For example, exhaustive degradation of cholesterol or other lipids may result in the accumulation of stable catabolic intermediates such as propionate which, if not detoxified, may reduce biological fitness (Chang et al., 2009). In addition, within the phagosome, MAP has to resist other microbicidal defense mechanism, e.g., changing levels of bivalent cations such as Fe, Cu, Zn (Soldati and Neyrolles, 2012). The predicted function of *mpt*D as part of an ABC transporter systems and its location on LSP14 suggest *mpt*D to be involved in ion homeostasis and imply that bivalent cations may serve as important regulators for balancing lipid metabolism of MAP during adaptation to the intracellular environment. Even though the precise function of *mpt*D is not yet known, our study exemplified that MAP-specific elements have a considerable role in MAP metabolism and give novel insights into their importance for metabolic adaptation of MAP to the host environment.

## Author contributions

Ralph Goethe, Gerald-F. Gerlach, and Jochen Meens designed the experiments; Gerald-F. Gerlach and Julia Heinzmann constructed the mutant, Boyke Bunk and Cathrin Spröer performed the mutant sequencing and alignments, Thorsten Meißner and Elke Eckelt characterized the mutant, Thorsten Meißner and Tina Basler performed the macrophage infections, Ralph Goethe and Siegfried Weiss designed the mouse infection experiments, Thorsten Meißner and Abdulhadi Suwandi performed the mouse infections, Sandra Trenkamp performed LC/MS-MS analysis; Walter M. R. Oelemann and Otto Holst performed the lipid profiling, Thorsten Meißner, Elke Eckelt, Jochen Meens, Ralph Goethe analyzed data; and Thorsten Meißner, Elke Eckelt, Gerald-F. Gerlach, and Ralph Goethe wrote the paper.

### Conflict of interest statement

The authors declare that the research was conducted in the absence of any commercial or financial relationships that could be construed as a potential conflict of interest.
