# Supplementary material for: The Mycobacterium avium ssp. paratuberculosis specific mptD gene is required for maintenance of the metabolic homeostasis necessary for full virulence in mouse infections
Source: Front Cell Infect Microbiol. 2014 Aug 14;4:110. doi: 10.3389/fcimb.2014.00110 (PMC4132290; doi:10.3389/fcimb.2014.00110)
Supplement: Supplementary file 1 [file DataSheet1.ZIP › Data Sheet 1/Datasheet 2.pdf]

Construction of allelic exchange substrate (AES) cosmid pMP1310

In order to generate an *mpt*D deletion in *Mycobacterium avium* subspecies *paratuberculosis* (MAP), two primer pairs were generated to amplify the 1039 bp upstream (LEFT; omptD1 / omptD2) and 1048 bp downstream (RIGHT; omptD3 / omptD4) flanking regions of the *mptD* gene. The obtained PCR products were cloned into the pCR2.1® TOPO vector using the TOPO TA cloning® kit, rendering plasmids pMP820 (LEFT) and pMP830 (RIGHT). The SpeI / XhoI fragment from pMP830 was cloned directionally into cosmid pYUB854 to generate pMP1301. Then, the AflII / XbaI fragment from pMP820 was cloned into AflII / XbaI restricted pMP1301 resulting in the new cosmid pMP1310 (Fig.S1). The inserted fragments of pMP1310 were controlled by DNA sequencing.

Construction of mycobacteriophage for *mpt*D deletion

To generate a specialized transducing mycobacteriophage, concatamers of purified phAE87 DNA were obtained by self-ligation. Next, pMP1310 and concatameric phAE87 DNA were restricted with *Pac*I, ligated, packaged into λ phage heads using GIGApack® II plus kit (Stratagen, La Jolla, California, USA) and transduced in *E. coli* HB101 according to the protocol of the manufacturer. The cosmid integrity of hygromycin resistant transductants was controlled by restriction digest and DNA sequencing. Correct cosmids were pooled and transformed into electro-competent *M. smegmatis* mc^2 155 cells. 100 µl aliquots of electroporated cells were mixed with 200 µl *M. smegmatis* mc^2 155, grown to an OD_{600} of 1.0, and 1.5 ml MB top agarose. These reactions were incubated on MBP agar plates for 5 days at 30°C and plaques were picked with sterile Pasteur pipettes and kept in MB buffer at 4°C overnight. 5 µl of each phage solution were mixed with 200 µl *M. smegmatis* mc^2 155 and 5 ml MB top agarose as described above, platted on two MBP plates and incubate in parallel at 42°C and 30°C, respectively, to confirm the temperature-sensitivity. One temperature sensitive phage (phAE111) was controlled for integrity of the AES by sequencing of the corresponding PCR fragments.

Phage Infection of *M. smegmatis,* titering of mycobacteriophage, confirmation of temperature-sensitive mycobacteriophage phenotype and the preparation of high titer mycobacteriophage stocks were essentially performed as described by Braunstein et al. (Braunstein, Bardarov, and Jacobs, Jr., 2002). Phage DNA was isolated in accordance to the protocol of Sambrook et al. (Sambrook and Russell, 2001).

Specialized transduction of MAP

The protocol was performed in according to Park and colleagues (Park *et al.*, 2008) with some modifications. MAP was grown to an OD_{600} to 1.0 in MB7H9 containing OADC and Tween 80®. The bacteria were singularized by vortexing with glass beads (2-3 mm diameter) for 15 min. Then, the culture was allowed to stand for 30 min on ice and the supernatant was transferred in a sterile 50 ml reaction tube. The bacteria were centrifuged at 7200 x *g* at RT for 10 min and washed three times in 30 ml MP buffer. Afterwards, the pellet was resuspended in 5 ml MP buffer and put on ice for 20 min to reduce clumps in the culture. Following, 1 ml of the supernatant was mixed with 1 ml of mycobacterial phage lysate in a 12 ml tube in a MOI of 10:1 and incubated for 4 h at 37°C. 2 ml MB broth containing 5 % OADC were added and the samples were incubated on a shaker (100 rpm) at 37°C for 24 h. Then, the culture was placed on ice for 30 min and 300 µl of the supernatant were plated on pre-warmed (37°C) MB 7H10 agar plates containing OADC and hygromycin B with concentration of 50 µg/ml and of 75 µg/ml. The plates were incubated for 8 weeks at 37°C and hygromycin resistant clones were tested for the successful *mpt*D deletion by PCR, real-time PCR and southern blot analysis.

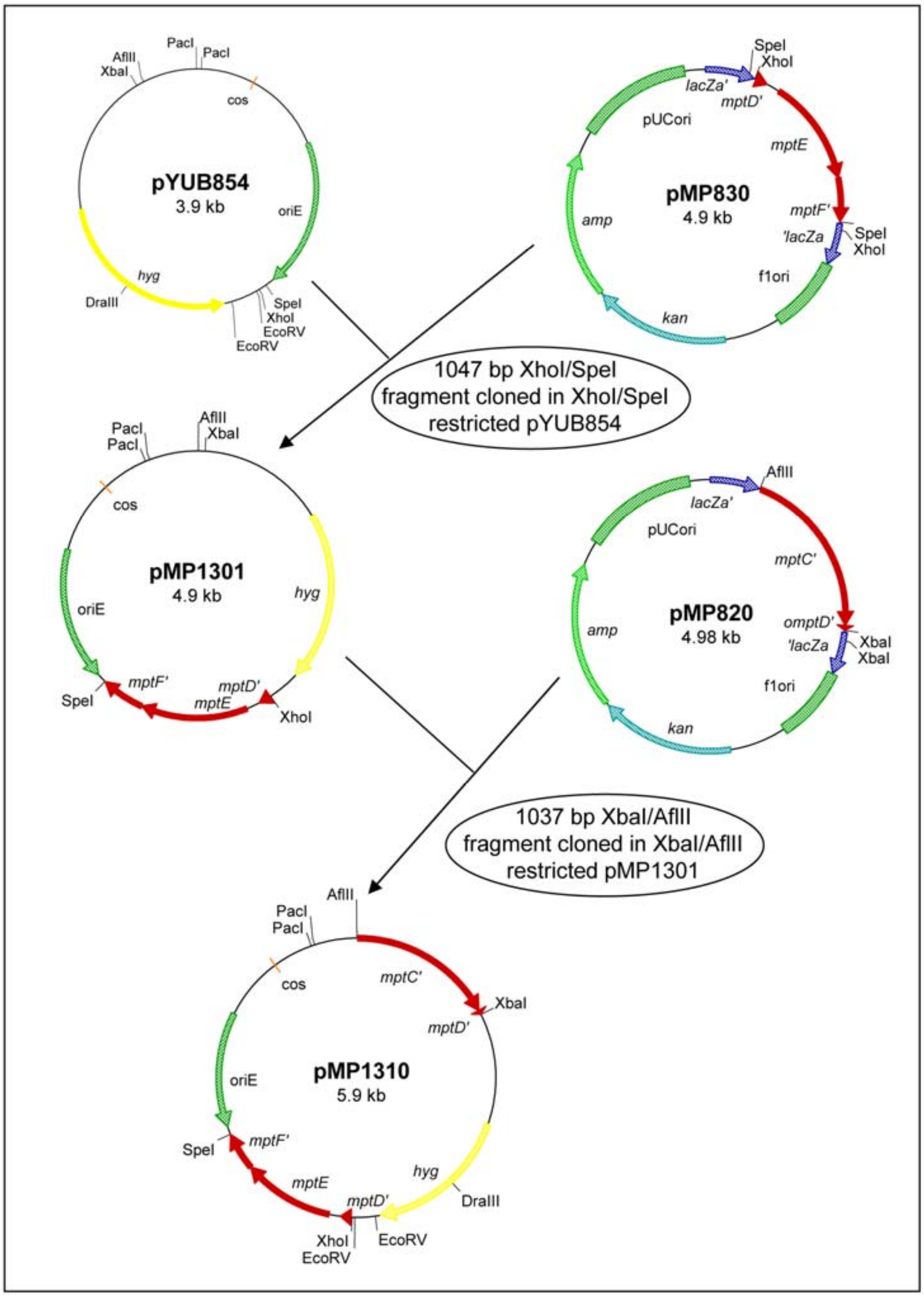

Scheme of the construction of the recombinant phasmid pMP1310. Arrows denote the direction of the respective reading frame in circular maps. *bla*, ampicillin resistance determinant, *kan*, kanamycin resistance determinant, *hyg*, hygromycin resistance determinant, *mpt*C´, *mpt*D’, *mpt*E and mptF’ indicate the positions of the respective ORFs in MAPwt (red). pYUB854: cosmid with λ-cos site (cos), pMP830: Topo vector with downstream element (*mpt*D´EF´), pMP820: Topo vector with upstream element (*mpt*C´D´), pMP1301: cosmid (pYUB854) with downstream element (*mpt*D´EF´), pMP1310: cosmid (pYUB854) with downstream element (*mpt*D´EF´) and upstream element (*mpt*C´D´).

Reference List

Bardarov,S., Bardarov S Jr, Pavelka,M.S., Jr., Sambandamurthy,V., Larsen,M., Tufariello,J., Chan,J., Hatfull,G., and Jacobs,J.W., Jr. (2002) Specialized transduction: an efficient method for generating marked and unmarked targeted gene disruptions in *Mycobacterium tuberculosis*, *M. bovis* BCG and *M. smegmatis. Microbiology* **148**: 3007-3017.

Bardarov,S., Kriakov,J., Carriere,C., Yu,S., Vaamonde,C., McAdam,R.A., Bloom,B.R., Hatfull,G.F., and Jacobs,W.R., Jr. (1997) Conditionally replicating mycobacteriophages: a system for transposon delivery to *Mycobacterium tuberculosis. Proc.Natl.Acad.Sci.U.S.A* **94**: 10961-10966.

Braunstein,M., Bardarov,S.S., and Jacobs,W.R., Jr. (2002) Genetic methods for deciphering virulence determinants of *Mycobacterium tuberculosis. Methods Enzymol.* **358**: 67-99.

Granger,K., Moore,R.J., Davies,J.K., Vaughan,J.A., Stiles,P.L., Stewart,D.J., and Tizard,M.L. (2004) Recovery of Mycobacterium avium subspecies paratuberculosis from the natural host for the extraction and analysis in vivo-derived RNA. *J.Microbiol.Methods* **57**: 241-249.

Jark,U., Ringena,I., Franz,B., Gerlach,G.F., Beyerbach,M., and Franz,B. (1997) Development of an ELISA technique for serodiagnosis of bovine paratuberculosis. *Vet.Microbiol.* **57**: 189-198.

Park,K.T., Dahl,J.L., Bannantine,J.P., Barletta,R.G., Ahn,J., Allen,A.J., Hamilton,M.J., and Davis,W.C. (2008) Demonstration of allelic exchange in the slow-growing bacterium *Mycobacterium avium* subsp. *paratuberculosis*, and generation of mutants with deletions at the pknG, relA, and lsr2 loci. *Appl.Environ.Microbiol.* **74**: 1687-1695.

Sambrook,J., Russell,D.W. (2001) Molecular Cloning: a Laboratory Manual, 3 nd edn. *Cold Sping Harbor, N.Y.: Cold Spring Harbor Laboratory.*

Snapper,S.B., Melton,R.E., Mustafa,S., Kieser,T., and Jacobs,W.R., Jr. (1990) Isolation and characterization of efficient plasmid transformation mutants of *Mycobacterium smegmatis*. *Mol.Microbiol.* **4**: 1911-1919.
