# Supplementary material for: The Mycobacterium avium ssp. paratuberculosis specific mptD gene is required for maintenance of the metabolic homeostasis necessary for full virulence in mouse infections
Source: Front Cell Infect Microbiol. 2014 Aug 14;4:110. doi: 10.3389/fcimb.2014.00110 (PMC4132290; doi:10.3389/fcimb.2014.00110)
Supplement: Supplementary file 1 [file DataSheet1.ZIP › Data Sheet 1/Figure S1.PDF]

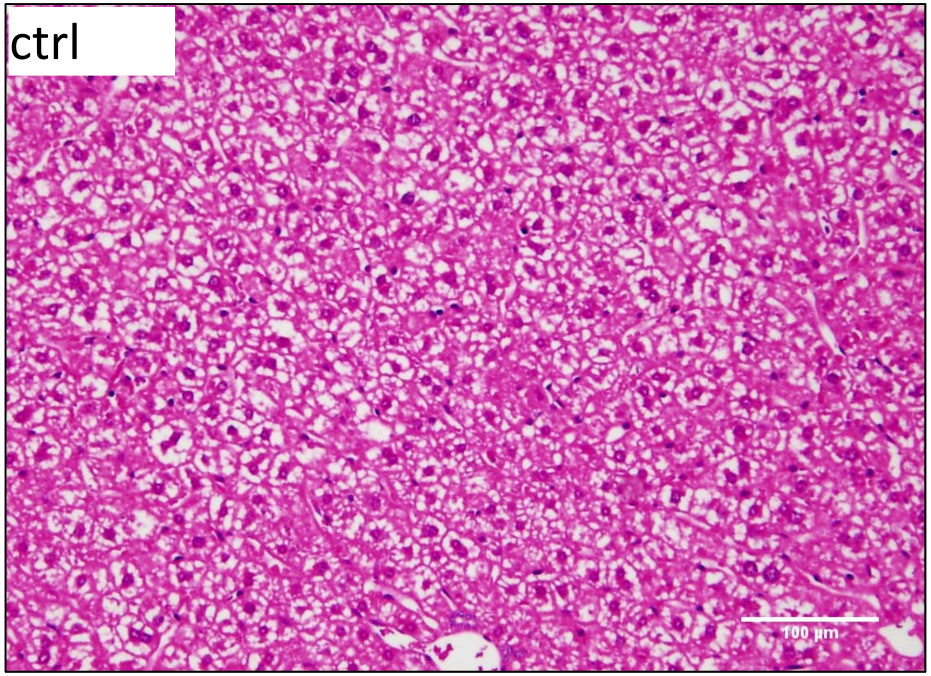

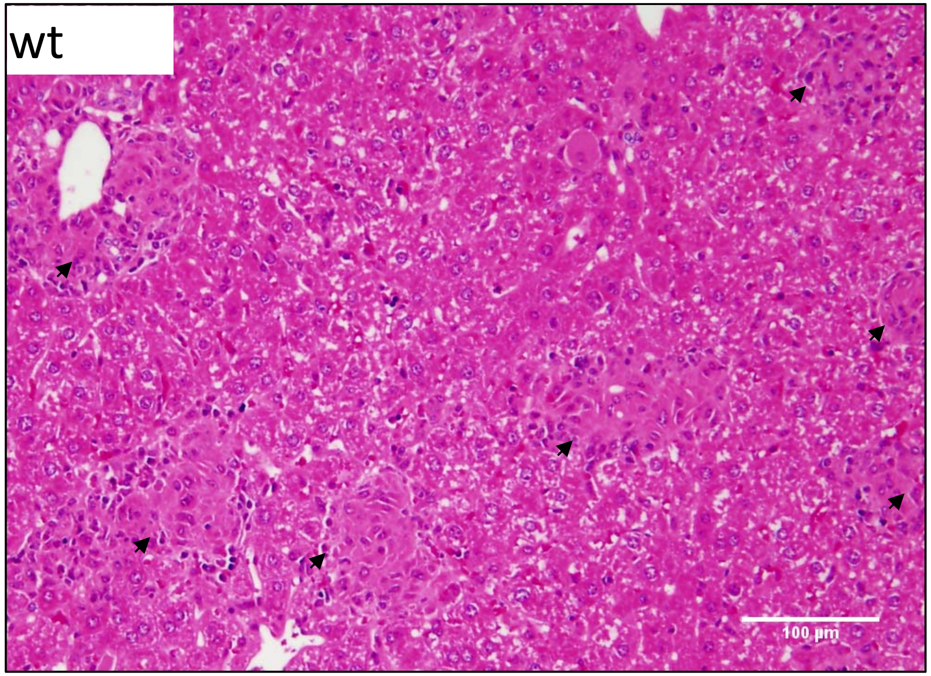

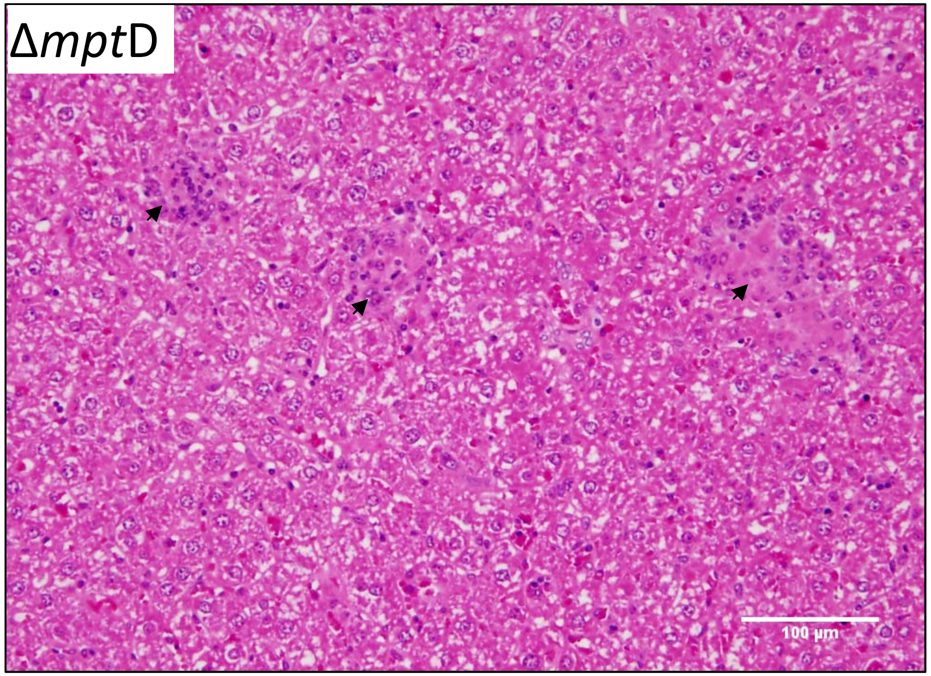

Suppl. Figure S1: Increased granuloma formation in mice infected with MAPwt. 8 weeks old C57BL/6 mice were challenged intraperitoneally with either MAPwt (wt) or MAPΔ*mpt*D (Δ*mpt*D) as described in Material and Methods. DPBS was used as a control (ctrl). Shown are representative pictures of HE-stained histological sections of the liver, magnification 100x. Black arrows mark the localization of granulomas.
